# Choosing appropriate prosthetic ankle work to reduce the metabolic cost of individuals with transtibial amputation

**DOI:** 10.1038/s41598-018-33569-7

**Published:** 2018-10-17

**Authors:** Kimberly A. Ingraham, Hwan Choi, Emily S. Gardinier, C. David Remy, Deanna H. Gates

**Affiliations:** 1 University of Michigan, Department of Mechanical Engineering, Ann Arbor, MI 48109 USA; 2 University of Michigan, School of Kinesiology, Ann Arbor, MI 48109 USA

## Abstract

Powered ankle prostheses have been designed to reduce the energetic burden that individuals with transtibial amputation experience during ambulation. There is an open question regarding how much power the prosthesis should provide, and whether approximating biological ankle kinetics is optimal to reduce the metabolic cost of users. We tested 10 individuals with transtibial amputation walking on a treadmill wearing the BiOM powered ankle prosthesis programmed with 6 different power settings (0–100%), including a prosthetist-chosen setting, chosen to approximate biological ankle kinetics. We measured subjects’ metabolic cost of transport (COT) and the BiOM’s net ankle work during each condition. Across participants, power settings greater than 50% resulted in lower COT than 0% or 25%. The relationship between power setting, COT, and net ankle work varied considerably between subjects, possibly due to individual adaptation and exploitation of the BiOM’s reflexive controller. For all subjects, the best tested power setting was higher than the prosthetist-chosen setting, resulting in a statistically significant and meaningful difference in COT between the best tested and prosthetist-chosen power settings. The results of this study demonstrate that individuals with transtibial amputation may benefit from prescribed prosthetic ankle push-off work that exceeds biological norms.

## Introduction

Individuals with transtibial amputation spend 10–30% more metabolic energy when walking compared to able-bodied individuals^1–3^. This observed increase in energy expenditure may be due to the fact that most ankle prostheses are passive-elastic devices, which store and release energy when in contact with the ground but cannot perform positive net work. In fact, passive-elastic prostheses only produce about an eighth the power of the intact gastrocnemius and soleus muscles^4^. This deficit has a significant impact on walking as the majority of the total mechanical power generated during the gait cycle comes from the ankle-foot complex^5–7^. As a result of decreased ankle power generation, people with amputation may put forth additional muscular effort from their residual limb or compensate with their intact limb to walk with passive prostheses^8^. Additionally, physical fitness may play an important role in determining the metabolic demands of individuals with amputation^9^.

To overcome these limitations of passive devices, various types of powered ankle prostheses have been developed^10–12^. These powered devices use actuators to deliver positive work to the user during the push-off phase, and can potentially alleviate the increased energetic demand that people with amputation experience during walking. Of these devices, only the BiOM powered ankle prosthesis (BionX, Bedford, MA) is currently commercially available. Investigating the efficacy of the BiOM in clinical trials has resulted in mixed outcomes. For walking speeds faster than 0.75 m/s, Herr *et al*.^10^ found a significant reduction in metabolic cost when individuals with transtibial amputation walked with a powered ankle prosthesis (BiOM) compared to passive, dynamic-response feet. Similarly, Esposito *et al*.^13^ found a 16% decrease in metabolic rate in highly active individuals using the BiOM compared to their prescribed dynamic-response feet. In contrast, Gardinier *et al*.^14^ found no significant differences in metabolic cost between individuals using the BiOM and dynamic-response prostheses. The mixed results of studies evaluating the efficacy of powered prostheses are likely related to a variety of factors, including different subject populations. In the study by Gardinier *et al*.^14^ individuals with the maximum functional classification level (K4) received an metabolic benefit from using the BiOM, while those with a lower functional classification (K3) did not. Correspondingly, the studies which demonstrated the largest reductions in metabolic cost were those that tested high-functioning active-duty military members^13^.

Another important factor when evaluating the BiOM’s impact on reducing metabolic energy expenditure is how the device is tuned; Esposito *et al*.^13^ suggested that near-optimal tuning of the BiOM is required to positively impact an individual’s metabolic cost. Of particular importance when tuning the BiOM are the *power settings*, which determine the amount of ankle work delivered by the device. The BiOM provides prothetists with a visual display to tune the power settings on the device. The prosthetist modifies the power settings until the ankle work delivered by the prosthesis approximates normative data of healthy ankle work at the subject’s preferred walking speed. Yet, it is still uncertain whether that amount of prosthetic ankle work is optimal to lower the user’s metabolic cost. In fact, a number of arguments could be made against this assumption: 1) the work produced by a prosthesis might not be delivered in full to the user’s center of mass, 2) a uniarticular prosthesis can never fully replicate the biarticular muscles in a human ankle, and 3) excess ankle work could be beneficial to compensate for other losses.

First of all, there is an open question regarding how much of the push-off work produced by a powered prosthesis is effectively delivered to the user. Prosthetic power may be lost in transmission due to residual limb deformation or the relative movement between the residual limb and the socket (i.e., pistoning)^15^. Compliant characteristics of foot cosmeses and shoes may also contribute to dissipating mechanical work of powered prostheses^16^. These additional losses might render the power delivered to the center of mass smaller than anticipated.

Furthermore, since all currently existing powered ankle prostheses are uniarticular, they can never fully replicate an intact human ankle. In particular, they cannot replace the function of the gastrocnemius, a biarticular muscle, which contributes to work at both the ankle and knee. This is important since both ankle plantarflexors (soleus and gastrocnemius) deliver energy to support the body and propel the center of mass forward^6^. In the absence of functional plantarflexor muscles, trunk support can be accomplished by energy return from a carbon fiber foot, but forward propulsion requirements may not be fully met^4^. Uniarticular actuation might further lead to insufficient energy transmission to initiate knee flexion during pre-swing. In order to propel the body and leg forward, other leg or hip muscles may compensate and thereby drive up metabolic cost, even though the supplied prosthetic plantarflexor power approximates biological norms. Thus, walking with prosthetic push-off work in excess of biological norms may provide not only body support but also propulsion, which more closely matches the function of intact plantarflexors.

Finally, there is some general evidence that walking with ankle work in excess of biological norms may reduce metabolic effort. Providing ankle power greater than the biological ankle has been shown to reduce metabolic cost in able-bodied subjects walking with exoskeletons^17,18^ and an ankle prosthesis adapter^19^. Simplified gait models suggest that ankle push-off work from the trailing limb can reduce the negative work performed through dissipation at heel contact by the leading limb^20^, yet recent human experiments with an ankle prosthesis adapter have not observed this effect^19,21^. A reduction in negative work and/or increase in push-off work may lower metabolic cost by reducing the required hip work of the stance leg during single support stance phase^3,22,23^. Although the exact biological mechanisms are not yet known, supplying prosthetic ankle work in excess of biological norms may be a useful strategy to compensate for increased effort in other places.

While this prior work suggests that increasing the amount of ankle work beyond the biological norms may be beneficial, we also believe that there may exist an upper bound with diminishing or negative return. For example, an excessive ankle plantarflexion moment can induce knee hyperextension^24^. In the non-amputated limb, the gastrocnemius and Achilles tendon restrain excessive knee extension during stance. In the amputated limb, however, the functional absence of these restraints may result in knee hyperextension with excessive prosthetic ankle push-off, thereby requiring other knee flexors to compensate and potentially increasing the user’s metabolic effort^13,25^. Additionally, the uniarticular function of the prosthesis may cause excess plantarflexion power to be re-distributed to the leg via the trunk and lead to increased hip power generation and increased knee power absorption^25^. As a result, these compensatory muscular efforts may contribute to increased metabolic cost. Due to the complexity of the human musculoskeletal system, there will likely be other examples of compensations that increase metabolic cost in response to excessive ankle power.

The goal of this study was to determine the effect of the BiOM’s ankle power setting on the metabolic cost of walking. We performed an experiment in which ten individuals with transtibial amputation walked on a treadmill wearing the BiOM powered ankle prosthesis, tuned to six different power settings. The power settings ranged from no power (0%) to the device’s maximum power setting (100%) in increments of 25%, and included the power setting chosen by the prosthetist during a clinical fitting session. We calculated subjects’ cost of transport (COT) during the steady-state portion of each condition. The BiOM records its net ankle work during each step, and we averaged this value over the last 30 steps of each condition to obtain the average net work. Our first hypothesis was that a subject’s energetically optimal power setting would be higher than the prosthetist-chosen power setting (i.e., the power setting chosen to approximate biological ankle kinetics). Our second hypothesis was that a subject’s energetically optimal power setting would not fall at 100% power (i.e., the maximum). We expected that the highest power setting would increase metabolic cost as the person is forced to absorb excess work with their musculoskeletal system.

## Results

**Figure 1:**
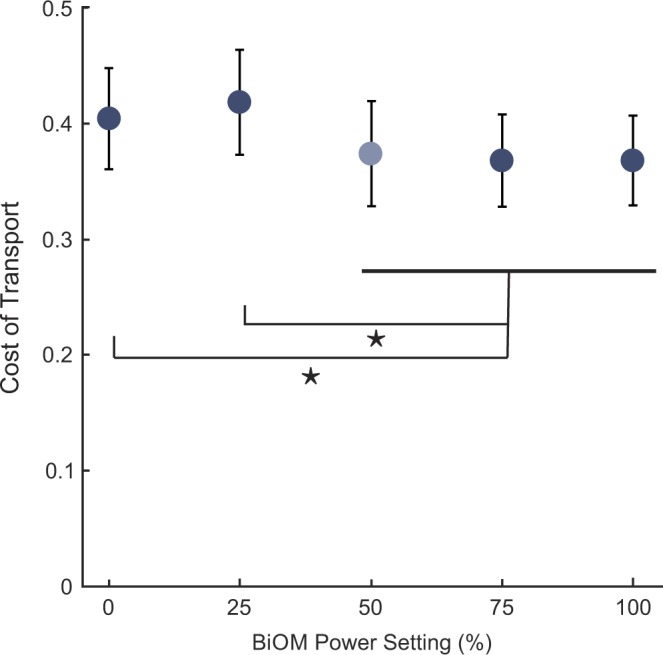
On average, subjects exhibited a lower cost of transport (COT) as power setting increased. Data points represent the group mean COT (dark blue: n = 9, light blue: n = 8, error bars: ±1 standard deviation). Stars (★) indicate a statistically significant difference between conditions (*p* < 0.05). For example, 0% had significantly higher COT than 50%, 75%, and 100% power conditions.

Overall, there was a significant main effect of BiOM power setting on cost of transport (COT) (*p* < 0.001). On average, power settings higher than 50% resulted in lower COT than 25% or 0% (Fig. 1). Post-hoc pairwise t-tests revealed significant differences between 0% power and 50% (*p* = 0.02), 75% (*p* < 0.001), and 100% (*p* = 0.01) power settings. There was also a significant difference between 25% power and 50% (*p* < 0.001), 75% (*p* < 0.001), and 100% (*p* < 0.001) power settings. There were no differences between the two lowest power settings (*p* = 0.16) and the three highest power settings (*p* > 0.57).

**Figure 2:**
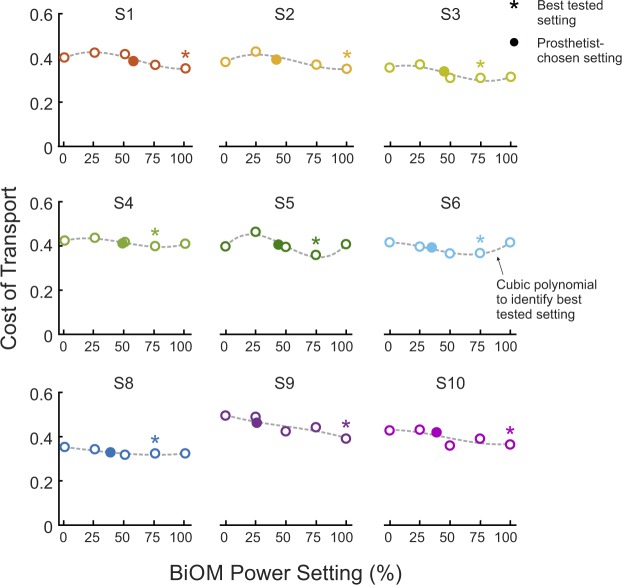
We observed large inter-subject variability in the relationship between power setting and the cost of transport (COT). Yet, for all subjects, the best tested power setting ($\ast$) was higher than the prosthetist-chosen power setting (•). Each subject’s best tested power setting was defined as the tested power setting closest to the minimum of the best fit cubic polynomial. This method was chosen to accommodate the noisy breath-by-breath measurements of metabolic cost and the sparse sampling of the parameter space. The third-order polynomials (dashed gray line) are presented for reference.

Examining subject-specific responses, we observed considerable variability between subjects (Fig. 2). Yet, for all subjects, the best tested power setting was higher than the prosthetist-chosen power setting. Five (5) of 9 subjects had best tested power settings at 75%, while the remaining 4 subjects were at 100%. Additionally, some subjects showed an increase in COT when the device provided some power (25%) compared to no power (0%).

**Figure 3:**
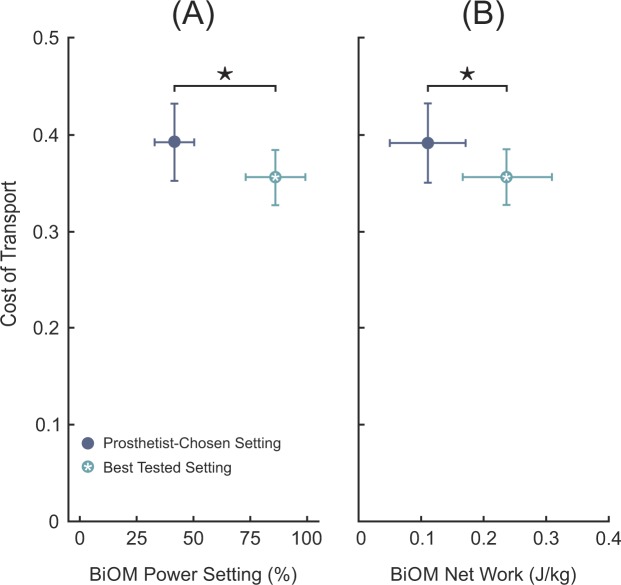
(**A**) On average, subjects walking with their best tested power setting had significantly lower cost of transport (COT) compared to walking with the prosthetist-chosen power setting (*p* < 0.001). The mean prosthetist-chosen power setting was 41.6% ± 8.7%; the mean best tested setting was 86.1% ± 13.2%. The star (★) indicates a significant difference in the magnitude of COT between the prosthetist chosen and best tested power settings. Error bars represent ±1 standard deviation in COT (vertical) and power setting (horizontal). (**B**) The corresponding mean net ankle work for the prosthetist-chosen and best tested conditions were 0.11 ± 0.06 J/kg and 0.24 ± 0.08 J/kg, respectively.

There was a significant difference in the magnitude of COT between the prosthetist-chosen setting (0.39 ± 0.04, mean ± SD) and best tested setting (0.36 ± 0.03) (Fig. 3). On average, this difference in COT was 0.04 ± 0.02, which corresponds to an 8.8% ± 4.6% reduction. The mean prosthetist-chosen power setting was 41.6 ± 8.7% (corresponding to mean ankle work of 0.11 ± 0.06 J/kg) (Fig. 3). The mean best tested power setting was 86.1 ± 13.2% (0.24 ± 0.07 J/kg). The mean participant-specific difference between prosthetist-chosen and best tested power settings was 44.6 ± 16.2% (0.12 ± 0.09 J/kg).

**Figure 4:**
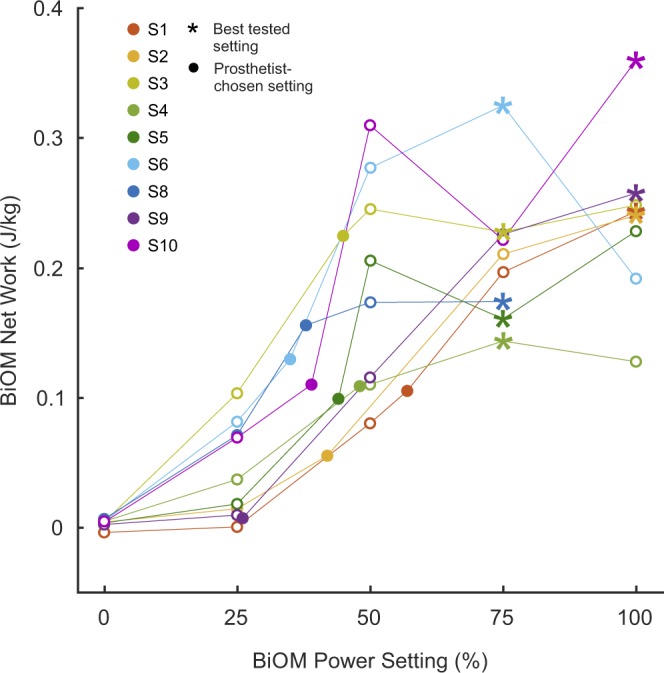
We found a linear correlation (Pearson’s *r* = 0.90 ± 0.07) between power setting and net ankle work for individual subjects. However, not all subjects exhibited a monotonically increasing relationship, and we observed a plateau in ankle work past the 50% power setting for some subjects. Filled circles (•) indicate the prosthetist-chosen power settings and corresponding net ankle work. Asterisks ($\ast$) indicate the subjects’ best tested power settings. Net ankle work for each condition was calculated as the mean of the ankle work from the last 30 steps for all conditions except Subject 4’s 75% condition, which was the average of 5 steps.

We also investigated how the power setting related to the net work performed by the BiOM. Overall, individual subjects showed a positive correlation (*r* = 0.90 ± 0.07) between power setting and net ankle work (Fig. 4). However, the relationship between power setting and ankle work was not strictly monotonic for all participants. Several subjects exhibited a plateau in ankle work as the power setting increased past 50%, and some even elicited less ankle work at higher power settings. Additionally, we observed variable responses between no power (0%) and a small amount of power (25%) for different subjects. Five (5) of 9 subjects elicited considerably more ankle work at the 25% condition than at the 0% condition (mean difference: 0.07 ± 0.02 J/kg, n = 5), while 4 other subjects elicited much more modest increases in ankle work between these conditions (mean difference: 0.01 ± 0.00 J/kg, n = 4). Similar trends were observed in the relationship between power setting and average peak ankle power (Supplementary Fig. S1).

**Figure 5:**
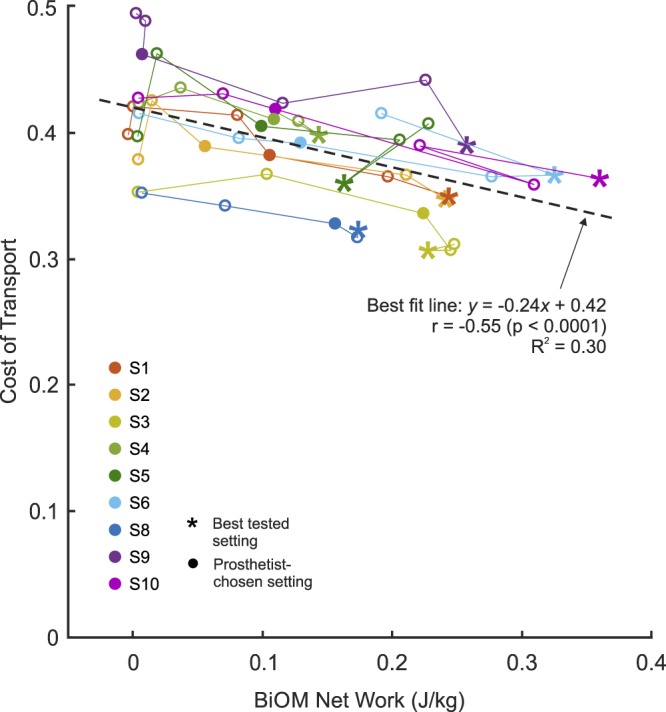
With all subjects pooled, there was a moderate linear correlation between cost of transport (COT) and net ankle work (Pearson’s *r* = −0.55, *p* < 0.0001). The best fit linear model (dashed line) resulted in *R*^2^ = 0.30. Individually, subjects exhibited a stronger linear relationship between COT and net ankle work (*r* = −0.82 ± 0.15, *R*^2^ = 0.69 ± 0.22). The majority of subjects’ best tested power settings corresponded to their maximum net ankle work. Starting from the left, colored lines connect increasing power settings for individual subjects. Filled circles (•) indicate the prosthetist-chosen power settings. Asterisks ($\ast$) indicate the best tested power settings. Net ankle work for each condition was calculated as the mean of the ankle work from the last 30 steps for all conditions except Subject 4’s 75% condition, which was the average of 5 steps.

Given that the relationship between BiOM power setting and net ankle work was not perfectly linear, we investigated the relationship between net ankle work and COT (Fig. 5). With all subjects pooled, a significant correlation between net ankle work and COT was identified (*r* = −0.55, *p* < 0.0001). The best fit linear model resulted in a coefficient of determination (*R*^2^) of 0.30. On an individual basis, subjects exhibited a stronger correlation between net ankle work and COT, with an average correlation coefficient of *r* = −0.82 ± 0.15. Six (6) of 8 subjects (excluding S8) had best tested power settings that corresponded to their maximum delivered ankle work (i.e., the asterisks in Fig. 5 lie farthest to the right). This did not coincide with the 100% power setting for Subjects 4 and 6, whose best tested power settings were 75%. The remaining two subjects analyzed (S3 and S5) had best tested power settings that did not correspond to their maximum ankle work. We do not have BiOM data from Subject 8 for the 100% condition, so we can not evaluate this relationship for this subject. Similar trends were observed in the relationship between average peak ankle power and COT (Supplementary Fig. S2).

## Discussion

In this study, we evaluated the influence of different prosthetic ankle power settings on users’ metabolic cost, using a commercially available powered prosthesis (BiOM). We hypothesized that 1) to minimize their energy cost, users would require a higher power setting than the power setting chosen by the prosthetist, which approximated the work of the biological ankle, and 2) the highest power setting (100%) would not be the optimal power setting to minimize metabolic cost.

In support of our first hypothesis, we found that the best tested power setting was higher than the prosthetist-chosen power setting for all subjects. Moreover, we found that on average, subjects walking with their best tested power setting significantly reduced their cost of transport (COT) by 0.04 ± 0.02 compared to walking with the prosthetist-chosen power setting. This change is larger than the within-day minimum detectable change (MDC) for COT reported by Davidson *et al*.^26^, which is 0.022. Therefore, we consider this difference a meaningful change and not likely due to measurement variation. On an individual basis, 6 of the 9 subjects analyzed had differences in COT greater than the MDC; the exceptions to this were Subject 1 (0.167), Subject 4 (0.012), and Subject 8 (0.005). The cost of transport calculated here is similar to values reported in other studies of people with transtibial amputation walking with the BiOM at similar walking speeds. In this study, the average COT for the prosthetist-chosen setting was 0.39 ± 0.04, while the average was 0.40 ± 0.05 in Gardinier *et al*.^14^ and 0.36 in Herr *et al*.^10^.

Since the prosthetist-chosen power setting was chosen to approximate the work done by a biological ankle during walking, our results suggest that individuals with transtibial amputation may require ankle work in excess of biological norms in order to reduce their metabolic effort. The biological net ankle work for non-amputee subjects walking at 1.25 m/s is approximately 0.1 J/kg^10,27^. On average, the best tested power setting in our study corresponded to net ankle work of 0.24 ± 0.07 J/kg, which is roughly double that of non-amputee subjects. As aforementioned, this finding is supported in studies conducted with able-bodied individuals wearing both exoskeletons^17,18^ and an ankle prosthesis emulator^19^. In contrast, a recent study by Quesada *et al*.^21^ did not show an effect of prosthesis work on the metabolic work rate of amputee subjects. Differences between our study and the study by Quesada *et al*. could have arisen from a variety of factors, including the functional level of the subject population and/or different prosthesis controllers. The subject cohort analyzed in our study comprised six K4 and three K3 individuals, while the cohort tested by Quesada *et al*. included six K3 and no K4 individuals. Gardinier *et al*.^14^ found that individuals with K4 functional level were significantly more likely to receive an metabolic benefit from the BiOM than those with a K3 level, which may partially explain the different outcomes of these studies. Additionally, the various prosthesis emulator work conditions tested in by Quesada *et al*. were generated by modifying the torque-angle relationship, and as such, the prosthesis work remained mostly constant between steps. In contrast, the BiOM device is controlled using a reflexive controller, which utilizes a neuromuscular model to command a torque at each step, and varies depending on how the user loads the prosthesis^10,28,29^. The reflexive controller and powered plantarflexion on the BiOM device are hypothesized to help users maintain balance, especially on variable terrain^29,30^. Similarly, Kim *et al*.^31^ demonstrated that a stabilizing controller which modulates ankle push-off work each step reduced metabolic cost and step width variability in able bodied-users walking with an ankle prosthesis emulator. The reflexive nature of the BiOM’s controller may have played an important role in the observed reduction of metabolic cost in the amputee subjects in our study, perhaps by reducing balance-related compensation efforts or allowing users to explore different assistance strategies by changing how they load the device step-to-step. Further studies will be required to determine the exact role that the reflex controller plays in reducing energy cost in prosthesis users.

The results of this study did not fully support our hypothesis that we would see an increase in COT at the highest power setting, indicative of too much ankle power. In fact, no significant group differences in COT were found between the 50%, 75%, and 100% conditions (corresponding to mean net ankle work of 0.19 ± 0.08 J/kg, 0.22 ± 0.05 J/kg, and 0.24 ± 0.07 J/kg respectively). When examining the data on a subject-specific basis, we saw that 5 of the 9 subjects analyzed had the best tested power setting at 75%. Yet, of these subjects, only Subjects 5 and 6 exhibited the anticipated trend of increased COT at 100% (both S5 and S6 increased their COT by 0.049 from 75% to 100%; the minimum detectable change (MDC) is 0.022). The other three subjects (S3, S4, S8) had changes in COT smaller than the MDC between 75% and 100%. The remaining three participants had their best tested settings located at the maximum (100%), which did not support our hypothesis. It is possible that these findings are also tied to the reflexive controller of the BiOM. Many subjects exhibited a plateau or a decrease in net ankle work past the 50% power setting, which also corresponded to little change in COT past this power setting. Accordingly, it appears that some subjects down-regulated the amount of work they received from the device at the higher power settings, most likely by not loading the device with their full body weight and exploiting the reflexive nature of the controller. As our experimental setup did not include force plates, we were unable to experimentally confirm this in the current study.

When we examined the relationship between net ankle work and COT (with all subjects pooled), we found a moderate linear correlation (*r* = −0.55). Yet, the best fit linear model only resulted in *R*^2^ = 0.30, which highlights the variability between subjects. On an individual basis, we found much stronger linear relationships between net ankle work and COT (*r* = −0.82 ± 0.15; *R*^2^ = 0.69 ± 0.22). Accordingly, most of the subjects tested (6 of 8, excluding Subject 8) had best tested conditions that corresponded to their maximum ankle work (see Fig. 5). For Subject 3, the difference in COT between the best tested condition (75%) and the condition with the maximum net ankle work (50%) was well below the minimum detectable change for COT (<0.01)^26^; this difference was much larger in Subject 6 (0.04). We could not complete this analysis for Subject 8, because we were unable to collect BiOM ankle work data during the 100% condition. Therefore, the power setting that maximized the ankle work delivered by the device was energetically optimal (or very close to energetically optimal) for nearly all participants in this study. Given this trend, however, it is also interesting to note that the net ankle work that corresponded to the best tested condition was quite variable between subjects, ranging from approximately 1.5 times (min: 0.14 J/kg) to 3.5 times (max: 0.36 J/kg) the work of a typical biological ankle.

The reflexive controller and corresponding variability in net ankle work that users produced between 0% and 25% power conditions could also explain why we saw an increase in COT for some subjects between these conditions. Although there were no significant group differences between these conditions, we observed an increase in COT between the 0% and 25% power conditions greater than the MDC for Subjects 1, 2, and 5 (mean difference: 0.04 ± 0.02, n = 3, see Fig. 2). Qualitatively, we noticed that these same subjects were those who elicited only a modest increase in net ankle work between 0% and 25% power conditions (mean difference: 0.01 ± 0.01 J/kg, n = 3, see Fig. 4). Similarly, those five subjects (S3, S4, S6, S8, S10) who elicited substantially more ankle work at 25% than at 0% (mean difference: 0.07 ± 0.02 J/kg, n = 5) exhibited a decrease or very small increase in COT between these conditions (mean difference: 0.00 ± 0.01). The final subject (S9), exhibited very little change in ankle work or COT between the 0% and 25% power conditions. These results seem to classify our subject cohort into two groups when a small amount of power was provided: those who took advantage of the power, and those who appeared to modify their behavior to avoid receiving power from the device and correspondingly increased their COT. If the work the device performed at this power setting was disruptive to the natural walking dynamics or balance of the subject, it is possible that they were “fighting” the device by increasing muscular co-contraction or adopting atypical compensatory gait mechanisms, which may have driven up metabolic cost for those participants^32^. Given our small sample size and the high level of breath-by-breath variability in the metabolic measurements, further analysis of additional biomechanical quantities (e.g., electromyography, inverse kinematics, spatiotemporal parameters) will be conducted to investigate these mechanisms in detail and to determine quantitative relationships, if any.

This study presents quantitative evidence regarding how users respond differently to various ankle power settings and exploit the BiOM’s reflexive controller in order to reduce their metabolic cost. There are several hypothetical reasons that users might adapt their gait to reduce the plantarflexion power they receive from the device at various power settings. Users could be actively off-loading the device at higher power settings because they feel uncomfortable or unstable, and resultant compensatory gait strategies or “fighting” the device could lead to higher energy consumption. However, it is also possible that users are subconsciously or passively adapting their interaction with the device in order to optimize a physiological objective function, such as minimal metabolic cost^33^, minimal impact forces^34^, or maximal stability^35^, among others. Further detailed analyses of additional biomechanical measures (e.g., electromyography, ground reaction forces) are required to elucidate the underlying causes for these observations. The results of this study provide some insight into additional elements of prosthetic control that may be necessary to reduce metabolic cost, beyond only the magnitude of ankle power delivered. The complex interactions between prosthetic control, metabolic cost, muscle activity, joint kinetics, stability, and patient satisfaction remain an important topic for continued future research in order to inform powered prosthetic ankle prescription and improve patient outcomes.

Our study is not without its limitations. First, there was a limited sample of participants, and due to the walking stamina necessary to complete the experiment, we only tested active, healthy individuals. People with higher levels of ambulatory function (K3-K4) are capable of walking with variable cadence and performing advanced ambulation tasks, and may be able to better adapt their gait in order to take advantage of power from the device. Additional studies with a modified protocol will be necessary to determine how these results extend to individuals with lower levels of ambulatory function. Second, it is possible that the five-minute acclimation time was insufficient for some participants to adjust to each power setting. Compared to other studies in which the users had hours^10^, multiple sessions^21^, or even weeks^13^ to acclimatize to a powered device and its conditions, the users in our study received less time to familiarize themselves with the power settings. Although the results from our study suggest that some participants were able to adapt to the power delivered from the device in this short amount of time, it is possible that some subjects may have required more time and/or specific training to fully adapt to each setting. Third, to prevent physical fatigue and respect time constraints, we tested participants in increments of 25%, which is a very coarse sampling of the parameter space, and may have limited our ability to identify the true energetically optimal power setting for all users. Due to device limitations, we could not test users past 100% power (0.24 ± 0.07 J/kg), so it is also possible that a more energetically favorable setting exists outside our tested range. As we do not know the exact physiological relationship between power setting and COT, we cannot currently extrapolate these results outside the tested range. Fourth, our experimental setup did not include an instrumented force treadmill so we were not able to experimentally validate the agreement between the BiOM’s step-by-step calculations of net ankle work and average peak ankle power and those values obtained through inverse dynamics. Future studies with the BiOM prosthesis that include an instrumented treadmill will further improve the generalizability of this study’s results. Finally, it is important to point out that our study was limited to evaluating the best power setting while participants walked on a level treadmill at a constant speed. It is likely that the optimal power setting would change when users walked at different speeds, at an incline or decline, or over variable terrain, so this study can not make universal claims about the optimal power setting for all tasks.

In conclusion, to minimize their metabolic energy consumption, subjects in this study required a higher power setting than the setting chosen by the prosthetist to approximate biological ankle kinetics. On average, the power setting setting which minimized energy cost corresponded to approximately double the net ankle work of the biological ankle. Furthermore, subjects walking at their best tested power setting exhibited a meaningful decrease in cost of transport compared to walking with their prosthetist-chosen power setting, which suggests that individuals may benefit metabolically from prescribed ankle power that exceeds biological norms. However, the varied responses between subjects also point to the need for subject-specific parameter tuning. As one solution, recent work has demonstrated the feasibility of automatically tuning assistive device parameters to minimize metabolic cost (i.e., body-in-the-loop optimization)^36–39^, and continued research in this area has the potential to impact clinical device prescription. Finally, subjects’ net ankle work was highly variable at different power settings, likely due to the reflexive controller of the BiOM and the user’s adaptation to the device. As such, future work should focus on quantifying the mutual adaptation of the human user and the device to inform the design of optimal powered prosthesis controllers for individuals with transtibial amputation.

## Methods

### Participants

Ten adult males with unilateral transtibial amputation participated in this study (Table 1). Subjects self reported their K-level and it was confirmed by the clinician. Potential subjects were excluded if they had a history of serious cardiovascular, neurological, respiratory, or visual problems, or were taking medications that might interfere with walking ability. Subjects were required to have the ability to walk for 30 minutes without a walking aid. Accordingly, all participants had a Medicare functional classification level of K3 or K4^40^, which corresponds to a moderate to high level of ambulatory function. Study procedures were reviewed and approved by the University of Michigan’s Medical School Institutional Review Board. The study was carried out in accordance with the approved protocol. All participants provided written informed consent prior to participation.

**Table 1:** Participant Demographics.

Participant	Age (years)	Sex	Weight^†^ (kg)	Height^†^ (m)	Leg Length^†,•^ (m)	K-Level
1	59	M	83.7	1.70	0.88	K3
2	24	M	88.2	1.81	0.97	K3
3	26	M	79.9	1.88	1.04	K4
4	60	M	123.8	1.84	0.92	K4
5	55	M	99.8	1.80	0.94	K3
6	32	M	87.3	1.85	0.98	K4
7*	27	M	120.2	1.78	0.96	K4
8	54	M	107.5	1.82	1.00	K4
9	53	M	73.9	1.70	0.89	K4
10	27	M	64.9	1.89	1.00	K4
Mean ± SD	41.7 ± 15.5	—	92.9 ± 19.5	1.80 ± 0.07	0.96 ± 0.05	—

^†^Measured while wearing the BiOM prosthesis.

^•^Average of left and right legs.

*Subject 7 was excluded from analysis due to prosthetic battery failures during collection.

### Prosthetic Fitting and Tuning

At the start of each session, all participants were fitted with the BiOM powered ankle prosthesis (BiOM T2 Ankle, BionX Medical Technologies Inc., Cambridge, MA), except for Subject 9 who was already a regular user of the BiOM. The same certified prosthetist fit each participant according to manufacturer’s recommendations. This process is described in detail in Gardinier *et al*.^14^. Briefly, the device was attached to the patient’s existing socket and pylon using standard attachments and then aligned. While wearing the device, we weighed each subject, and measured their height (the distance from the floor to the crown of head), and the length of both legs (the distance from the greater trochanter to the floor). Each subject’s reported leg length is the average of the left and right legs (Table 1).

**Figure 6:**
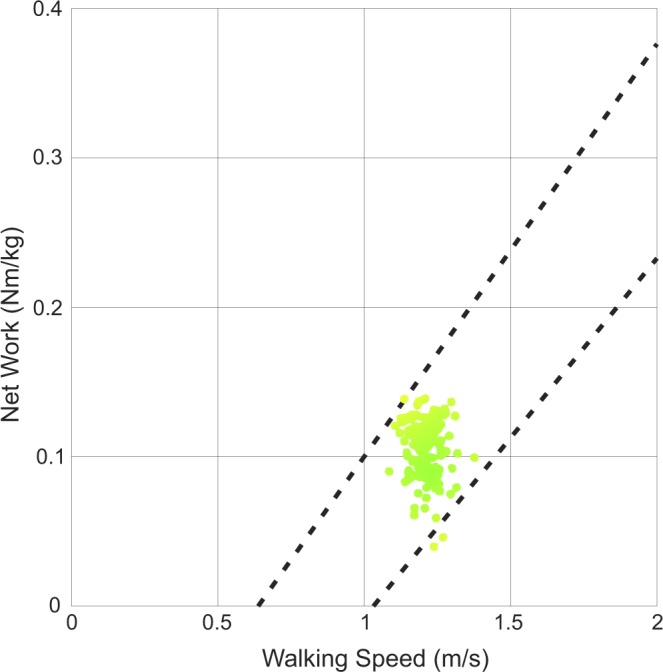
An example of the real-time display used to tune the actuation settings of the BiOM prosthesis (adapted from the BiOM user’s manual^49^). As the subject walks, a dot appears on the screen that indicates the work done by the prosthesis at the user’s chosen walking speed. The prosthetist modifies the power setting of the device until the dots fall into the ±95% confidence interval for healthy biological net ankle work (dashed lines) and the patient is satisfied with the device performance.

Next, the device was powered on and participants walked back and forth down a 20-meter-long hallway while the prosthetist increased the amount of net ankle work delivered by the device using a Bluetooth-enabled tablet computer. With each step, the tablet displays a dot corresponding to the net ankle work done by the prosthesis at the participant’s chosen walking speed (Fig. 6). This dot is green if it falls within the ±95% confidence interval of normative data for net ankle work at that walking speed; net ankle work outside of this range is displayed as orange or red. It is important to note that while the prosthetist is able to see the net work performed by the device with each step, he or she does not have direct control over this parameter. Rather, the prosthetist tuning the device can change the power *setting* of the device, which ranges from 0% power to 100% power in increments of 1%. In our study, the prothetist adjusted the power setting until the net ankle work fell within the desired range and the walking looked smooth and felt comfortable to the participant. After the power setting, adjustments were made to other settings, such as the timing of the power onset. Participants were given 30 minutes to acclimate to the device once initial fitting was complete. During this time, the prosthetist fine-tuned the actuation settings to best fit the comfort of the user as necessary. The final power setting is subsequently referred to as prosthetist-chosen power setting. Participants’ other settings (e.g., timing) were held constant throughout the experiment. A complete list of all the BiOM settings for each participant is provided in Supplementary Table S1.

### Experimental Protocol

**Figure 7:**
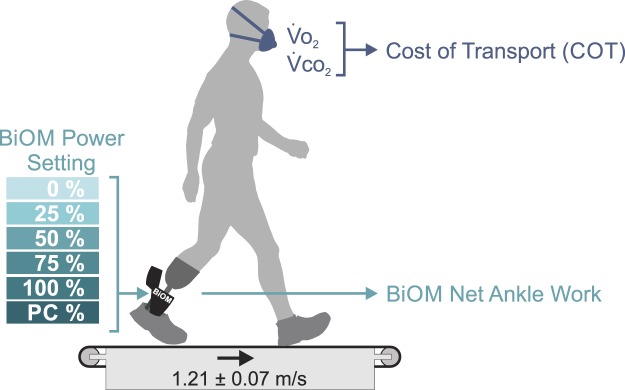
In this experiment, 10 transtibial amputees walked on a treadmill wearing the BiOM powered ankle prosthesis with 6 different power settings: 0%, 25%, 50%, 75%, 100%, and the parameter setting chosen by the prosthetist during fitting (prosthetist-chosen, PC). We measured metabolic energy expenditure using indirect calorimetry and calculated the cost of transport (COT) from metabolic cost and treadmill speed. Treadmill belt speed was normalized to leg length, and determined using a Froude number of 0.16, which corresponds to the typical preferred walking speed for individuals with transtibial amputation^50^. In our experiment, subjects walked at an average speed of 1.21 ± 0.07 m/s. We also collected net ankle work from the BiOM for each step during all conditions.

After the initial acclimatization period, participants completed six different conditions in a randomized order while walking on a treadmill wearing the BiOM. Each of these conditions corresponded to a different ankle power setting. The conditions tested were 0%, 25%, 50%, 75%, 100% power, and the prosthetist-chosen power setting (Fig. 7). During all treadmill walking trials, participants wore a support harness (Likorail, Hill-Rom, Chicago, IL), which prevented them from falling in case of loss of balance, but did not provide any upward force or body weight support. Treadmill belt speeds were normalized according to a Froude number of 0.16 to scale belt speed to each participant’s mean leg length^41^; the average walking speed for all participants was 1.21 ± 0.07 m/s.

At the beginning of each session, subjects were fit with a lightweight portable metabolic system (K4b^2^, Cosmed, Rome IT) consisting of a mask covering the nose and mouth and a portable unit attached to a harness. The metabolic system measured the rate of oxygen consumption ($\dot{V}{O}_{2}$) and carbon dioxide production ($\dot{V}C{O}_{2}$). Testing was generally performed first thing in the morning, and all participants were instructed to fast for at least two hours prior to arriving at the laboratory, as time of day and food consumption can affect one’s basal metabolic rate^42,43^. Participants were initially asked to remain seated for a period of at least 10 minutes to establish a baseline metabolic energy cost for each individual. We subsequently recorded participants’ metabolic energy cost during all treadmill walking trials. Additionally, we recorded net ankle work and peak ankle power from each step of the prosthesis via the tablet computer for all walking conditions. The BiOM calculates ankle joint work from its on-board sensors as the integral of ankle torque-angle curve; it calculates peak ankle power by multiplying the measured ankle angular velocity with the ankle torque^10^. Ankle torque is calculated as the combined torque of the motor and the estimated torque of the carbon fiber foot^44^.

Data were collected during one experimental session for most participants, but two participants (S1 and S5) required two sessions to complete all six conditions due to time constraints or physical fatigue. For these two subjects, we compared seated resting $\dot{V}{O}_{2}$ measurements from both experimental sessions against the between-day minimum detectable change threshold to assess the consistency of metabolic measurements across days. These between-day differences were less than the minimum detectable change threshold^26^, and were therefore regarded as within the expected variation and not meaningful. As such, we combined the data collected across both days for analysis. Two trials were excluded due to BiOM battery failure in the middle of the walking trial, which cut the trials short (S2: 50% condition, S8: 100% condition). We were unable to re-collect these trials due to participant fatigue and time constraints. The BiOM battery also died during Subject 4’s 75% condition, but we were able to collect 5 steps at this power setting at the end of the session. During the data collection session for Subject 7, both BiOM batteries became defective and would not charge. As we were only able to collect complete data for 2 of 6 conditions, this participant’s data was excluded from all analyses.

### Data Analysis

Participants walked with each power setting for a minimum of five minutes in order to reach a steady-state oxygen consumption level, and then three minutes of steady-state data were collected and subsequently analyzed. We confirmed that the last three minutes of recorded breath measurements for each walking condition were indeed at a steady state by testing that they met three criteria. These criteria were 1) average $\dot{V}{O}_{2}$ and 2) average $\dot{V}C{O}_{2}$ had less than 10% variability between minutes^45,46^, and 3) the average respiratory quotient (RER) was between 0.7 and 1.0^47^. We estimated metabolic power from the steady-state $\dot{V}{O}_{2}$ and $\dot{V}C{O}_{2}$ measurements using the Brockway equation^48^.

To generalize energy expenditure across different walking velocities between participants, we calculated the cost of transport (COT) by dividing the metabolic power by the walking velocity and body weight while wearing the BiOM device. COT is a dimensionless quantity. We averaged the last 30 steps of net ankle work data and peak ankle power data from the BiOM to obtain the average net ankle work and average peak ankle power for each condition, and normalized these quantities by body mass (including the weight of the prosthesis). In the case of Subject 4 walking at the 75% condition, they are the average value of the five collected strides.

### Statistical Analysis

We use a generalized linear model to determine the effect of BiOM power setting (0%, 25%, 50%, 75%, 100%) on COT. Power was a fixed factor, while subjects was a random factor. Post-hoc paired t-tests were used to explore significant differences between power settings. To determine a subject’s energetically optimal power setting, we fit a third order polynomial to each subject’s cost of transport data from the six power conditions (including the prosthetist-chosen). We then identified the minimum of each subject’s third order polynomial, and chose the tested power setting (0%, 25%, 50%, 75%, 100%, or prosthetist-chosen) closest to the minimum; this power setting is called the *best tested* power setting. This method of identifying the best tested power setting was chosen to minimize post-hoc selection bias, given the high level of breath-by-breath variability in the metabolic measurements and the sparse sampling of the parameter space. We used paired t-tests to evaluate the differences between the prosthetist-chosen and best tested power settings. Statistical comparisons were made using SPSS (IBM., Chicago, IL) with a level of significance of 0.05. Additionally, we calculated Pearson’s correlation coefficients between COT, average net ankle work, and BiOM power setting using MATLAB (MathWorks, Natick, MA).

## Electronic supplementary material

Supplementary Information

Dataset 1

## Data Availability

The data that support the findings of this study are available from the corresponding author (gatesd@umich.edu) upon reasonable request. Source data for Figs 1–5 are provided with the paper as supplementary information.
