# Supplementary material for: Choosing appropriate prosthetic ankle work to reduce the metabolic cost of individuals with transtibial amputation
Source: Sci Rep. 2018 Oct 17;8:15303. doi: 10.1038/s41598-018-33569-7 (PMC6193045; doi:10.1038/s41598-018-33569-7)
Supplement: Supplementary file 1 — Supplementary Information [file 41598_2018_33569_MOESM1_ESM.pdf]

Choosing appropriate prosthetic ankle work to reduce the metabolic cost of individuals with transtibial amputation

Kimberly A. Ingraham[1,+], Hwan Choi[2,+], Emily S. Gardinier[2], C. David Remy[1], and Deanna H. Gates[2,*]

[1]University of Michigan, Department of Mechanical Engineering, Ann Arbor, MI, 48109, USA
[2]University of Michigan, School of Kinesiology, Ann Arbor, MI, 48109, USA
[*]Corresponding Author: gatesd@umich.edu
[+]these authors contributed equally to this work

Supplementary Information

Subject	Stiffness (%)	Power at Fast Cadence (%)	Power at Slow Cadence (%)	Power Sensitivity (%)	Timing at Fast Cadence (%)	Timing at Slow Cadence (%)	Cadence Range (%)
S1	12	57	40	0	44	20	47
S2	45	42	41	0	44	44	49
S3	65	45	24	0	44	16	42
S4	50	48	25	0	44	16	81
S5	63	44	33	0	44	20	57
S6	81	35	25	0	44	16	45
S8	75	38	28	0	66	30	66
S9	0	26	26	0	44	16	36
S10	75	39	20	0	65	20	28

Supplementary Table S1. Prosthetist-Chosen BiOM Settings.

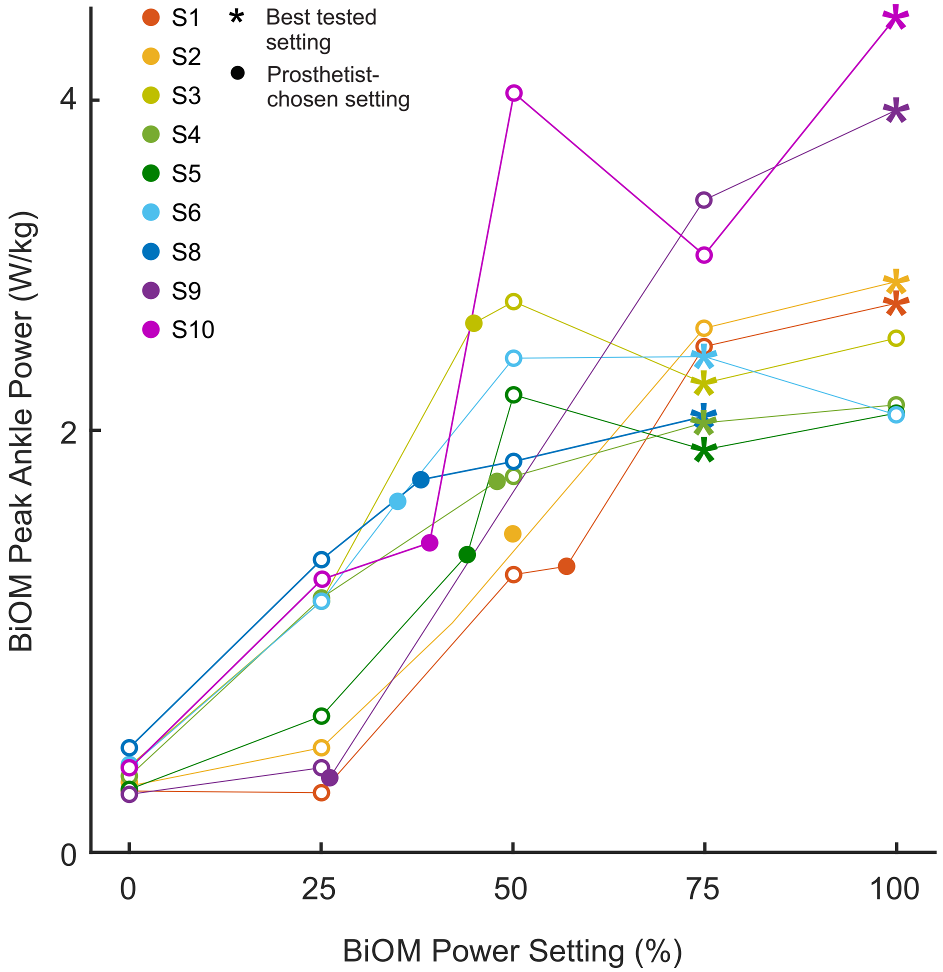

Supplementary Figure S1. We found a linear correlation ($r = 0.90 \pm 0.07$) between power setting and average peak ankle plantarflexion power for individual subjects. However, not all subjects exhibited a monotonically increasing relationship, and we observed a plateau in ankle power past the 50% power setting for some subjects. Filled circles (●) indicate the prosthetist-chosen power settings and corresponding ankle power. Asterisks ($*$) indicate the subjects' best tested power settings. Average peak ankle power for each condition was calculated as the mean of the peak ankle power data from the last 30 steps for all conditions except Subject 4's 75% condition, which was the average of 5 steps.

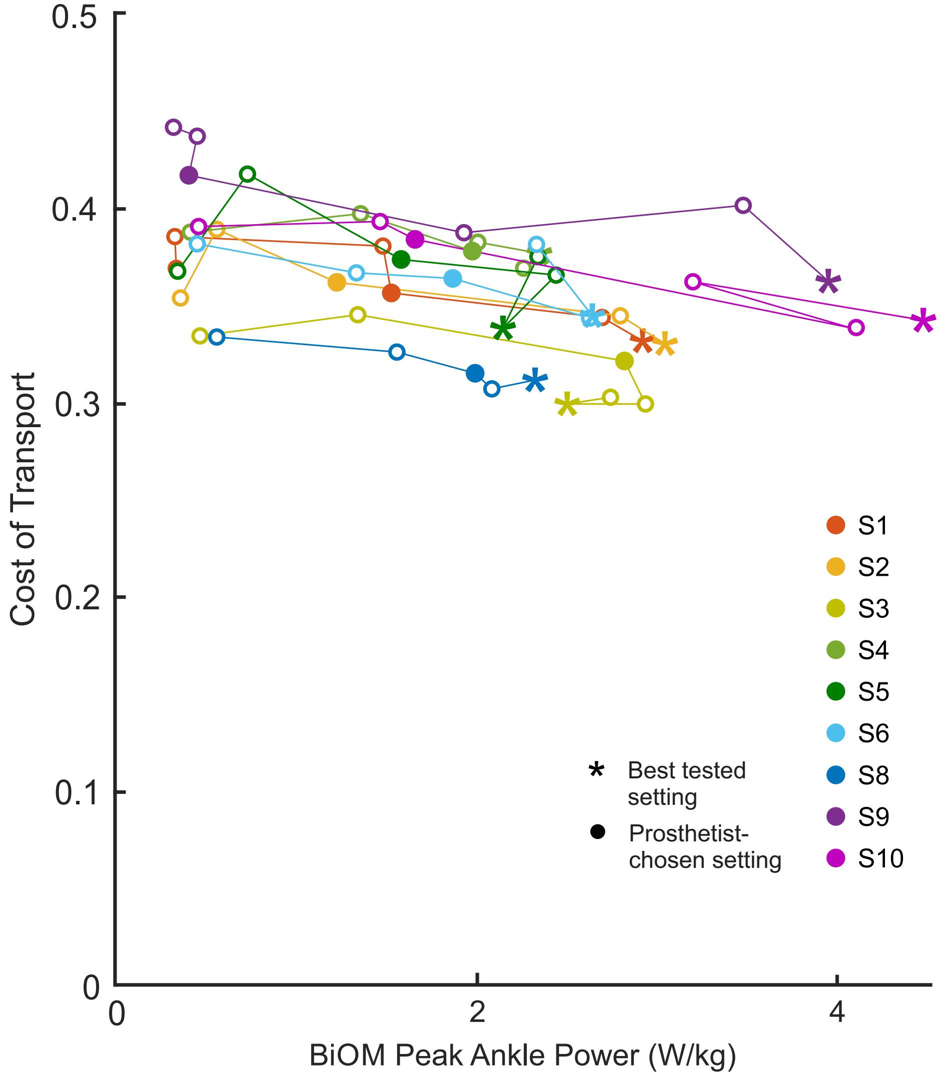

Supplementary Figure S2. Subjects exhibited a moderately strong linear correlation between cost of transport (COT) and average peak ankle power ($r = -0.77 \pm 0.15$), with large inter-subject variability. The majority of subjects' best tested power settings corresponded to their maximum ankle power. Starting from the left, colored lines connect increasing power settings for individual subjects. Filled circles (•) indicate the prosthetist-chosen power settings. Asterisks ($*$) indicate the best tested power settings. Average peak ankle power for each condition was calculated as the mean of the peak ankle power data from the last 30 steps for all conditions except Subject 4's 75% condition, which was the average of 5 steps.
